# Finite Element Numerical Simulation and Repair Process of Laser Cladding Repair of Surface Cracks on Mechanical Parts

**DOI:** 10.3390/mi15121428

**Published:** 2024-11-27

**Authors:** Shuangyang Yu, Qi Chong, Jianzhu Zhou, Youwen Yang, Hua Li

**Affiliations:** School of Mechanical and Electrical Engineering, Jiangxi University of Science and Technology, Ganzhou 341000, China9120180034@jxust.edu.cn (J.Z.)

**Keywords:** gear crack, laser cladding, numerical simulation, finite element analysis, temperature field, stress filed

## Abstract

This study focuses on the planetary gear reducer and employs ANSYS 13.0 software to perform thermo-mechanical coupled simulations for the laser cladding repair process, aiming to address gear failure caused by cracks. The optimal theoretical repair parameters were determined based on temperature and stress field analyses, and performance testing of the cladding layer was conducted to validate the feasibility of the selected parameters. The results suggest that a laser power of 140 W and a scanning speed of 8 mm/s represent the optimal theoretical parameters for the laser cladding repair of the gear workpiece. Tensile strength tests revealed that the cladding layer’s maximum tensile strength reached 1312.80 MPa, which was 1.22 times higher than that of the substrate material. Additionally, the wear resistance tests indicated that the wear loss of the cladding layer under the optimized parameters reduced from 9.3 mg for the base material to 0.5 mg, demonstrating excellent wear resistance. Thus, the mechanical properties of the cladding layer were significantly enhanced compared to the base material under these theoretical process parameters.

## 1. Introduction

Planetary gear reducers are critical components in mechanical transmission systems. However, since planetary gears must endure alternating, impact, and contact stress during operation, they often experience wear and fractures, compromising transmission efficiency and potentially causing machine failure in severe cases [1,2]. Conventional welding processes involve high temperatures, often resulting in issues like thermal distortion and material oxidation. High temperatures also deteriorate material properties, making it difficult to meet the demands of high-precision production. In contrast, the laser cladding repair process provides rapid heating with minimal heat input, resulting in limited thermal distortion of the base material.

As remanufacturing technologies evolve and improve, the repair and remanufacture of failed gears have significantly improved [3,4]. Laser cladding, as an advanced surface modification technique, offers excellent metallurgical bonding between the substrate and cladding layer, minimizes the heat-affected zone, and yields improved cladding properties with easier post-repair processing [5,6]. Therefore, laser cladding technology introduces an innovative approach to enhancing the quality of gear repair [7,8]. However, due to the complexity of the thermodynamic changes in laser cladding, experimentally optimizing process parameters is both cost-intensive and time-consuming [9,10]. A combination of numerical simulation and experimental validation enables observation of the thermo-mechanical coupling in the laser cladding process, mitigating these drawbacks [11].

The failure of large mechanical equipment components is a prevalent issue in the mechanical industry. Enhancing the surface performance of such components can improve their resistance to adverse working conditions and extend their service life. This study focuses on planetary gear reducers, employing ANSYS Workbench finite element software for the thermo-mechanical coupled simulation of the laser cladding repair of failed gears. By analyzing the temperature and stress fields under various process parameters, the optimal theoretical repair process was determined. Alongside laser cladding experiments, the mechanical properties of the formed cladding layer were analyzed to confirm the feasibility of the results, offering theoretical and experimental foundations for the laser cladding repair of planetary gear reducer cracks [12,13]. Failure of large mechanical equipment components is the most common problem in the field of mechanical industry; improvement of the surface performance of mechanical components can advance its adaptability to poor working conditions and resistance, delaying its service life. The results of this study can also provide a reference basis for the repair of large mechanical equipment parts.

## 2. Finite Element Numerical Simulation Model for Laser Cladding of Gear Cracks

### 2.1. Basic Assumptions of the Model

As laser cladding involves the interaction of multiple physical fields, numerical simulations cannot fully account for all factors involved in the process. Therefore, within a reasonable margin of error, the following assumptions about the material and boundary conditions are made [14]:

(1) The substrate and cladding layer materials are isotropic, and all process parameters except for thermal conductivity and specific heat capacity are assumed to remain constant with respect to temperature.
(2) The influence of molten pool flow and thermal radiation on the temperature field is ignored.
(3) The latent heat of phase transformation is not considered in the temperature field.
(4) The initial temperature of the model is set to 22 °C.

### 2.2. Thermal Physical Properties of Materials

**Figure 1:**
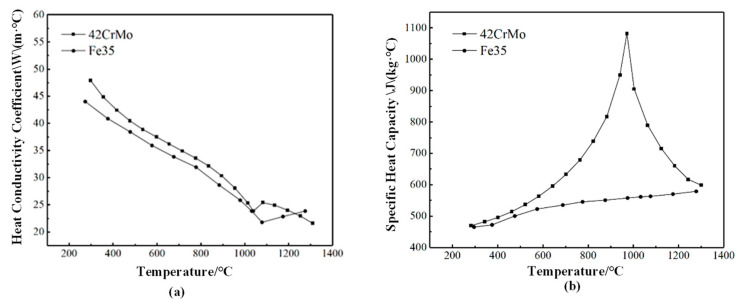
(**a**) is the thermal conductivity curve with temperature, and (**b**) is the specific heat capacity curve with temperature.

The base material used in this study is 42CrMo alloy steel, and Fe35 is selected as the filler material for the laser cladding experiment in accordance with the material selection guidelines [15,16]. As shown in Table 1, iron-based alloy powder exhibits excellent wear resistance and is suitable for parts requiring localized wear protection. As shown in Figure 1a, the hardness and wear resistance of the coating can be enhanced by adjusting the content of alloying elements, making it suitable for the repair and protection of gear surfaces, cast iron components, and rails. As shown in Figure 1b, although its melting point is high, the performance of its fused coating layer can be improved by modifying the composition. Additionally, iron-based alloy powder demonstrates excellent processability and is easy to apply through spraying techniques. The addition of appropriate amounts of Cr and Mo elements enhances its self-melting and spray welding performance, making it suitable for a wide range of 42CrMo workpiece repairs.

### 2.3. Geometric Model and Mesh Generation

**Figure 2:**
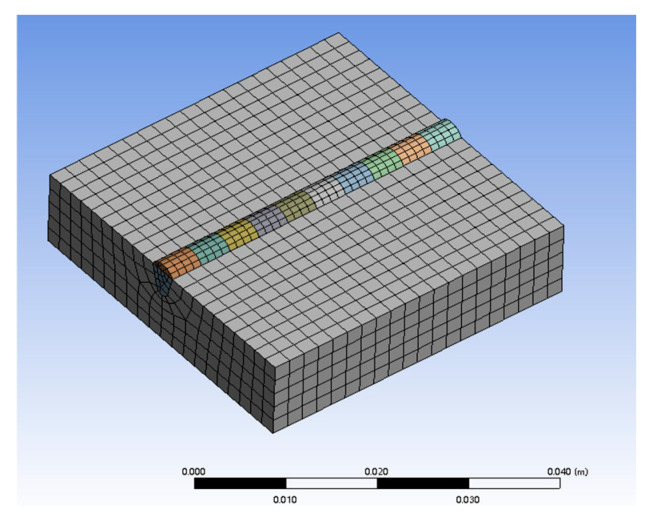
Grid division results.

The gear is modeled as a 40 × 40 × 10 mm 42CrMo substrate. In the temperature field simulation, 8-node hexahedral elements (SOLID70) are used, while for the stress field, the thermal elements are converted to structural elements (SOLID186). To improve computational precision and efficiency, mesh refinement is applied to the cladding layer and bonding area with a cell size of 1 mm, while the region far from the laser scan has a cell size of 2 mm. After meshing using the geometry size adjustment method, the model consists of 2960 elements and 16,043 nodes in total. The mesh generation results are shown in Figure 2.

### 2.4. Heat Source Model

**Figure 3:**
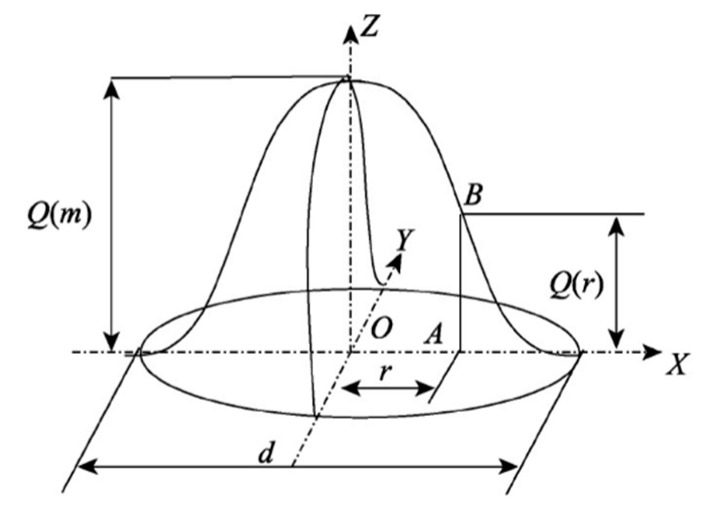
Diagram of a normal Gaussian heat source.

The heat source is closely linked to energy distribution and directly impacts the accuracy of the temperature field simulations. As shown in Figure 3, given that both the substrate and cladding powder simultaneously absorb laser energy in the coaxial powder feed laser cladding process and the laser beam moves across the substrate surface, a moving Gaussian heat source model is used [17,18].

(1) $$q\left(x,z,t\right)=\frac{3P}{\pi r_{0}^{2}}e^{\frac{-3\left({\left[x+v\left(\tau -t\right)\right]}^{2}+y^{2}\right)}{r_{0}^{2}}}$$

In the formula: *q*(x,z,t) is the heat flux density at a distance from the center of the laser spot (W/m²); *P* is the effective laser power (W); r₀ is the radius of the Gaussian heat source distribution (m); x, y is the radiation radius of the laser heat source in the *x* and *y* directions (m); v is the movement speed of the laser heat source (m/s); and τ is the time factor (s).

### 2.5. Initial Conditions and Boundary Conditions

In the temperature field, the initial temperature of the model and the convective heat transfer coefficient are required to be defined. In the solid model in this study, the substrate is modeled as unrepeated, with both the initial and ambient temperatures set to 22 °C. The convective heat boundary conditions involve defining a convective heat transfer coefficient between the substrate surface and the air. Furthermore, during the interlayer cooling process, the convective heat transfer coefficient between the surface of the first cladding layer and the air is incorporated [19,20]. This coefficient varies with temperature, enhancing the precision of the model’s computational results. In the stress field, the temperature field results are applied as loads. Given that the workpiece needs to be placed flat and fixed during the laser cladding process, a fixed support is added to the model’s bottom surface to limit the displacement degrees of freedom [21,22].

## 3. Finite Element Numerical Simulation Results Analysis of Laser Cladding for Gear Cracks

### 3.1. Analysis of Numerical Simulation Results for the Temperature Field

#### 3.1.1. Effects of Different Process Parameters on Temperature Field Distribution

**Figure 4:**
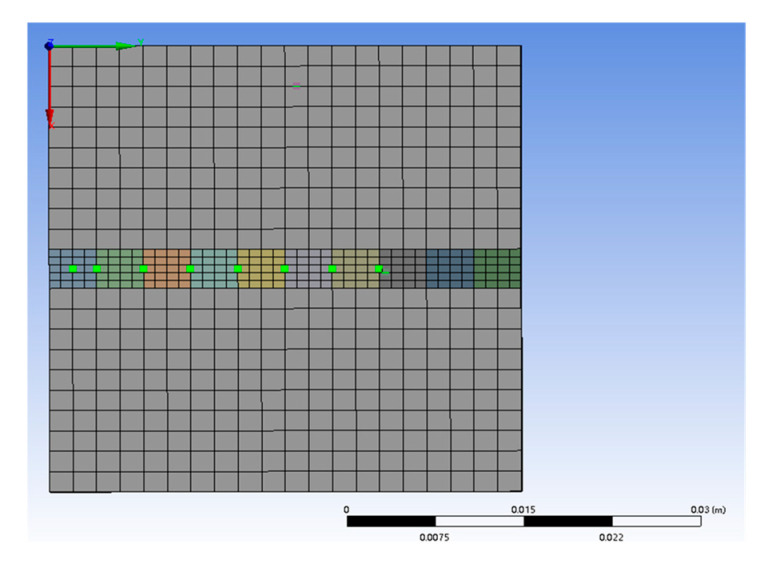
Node selection on the transverse surface of the cladding layer.

Due to laser beam irradiation, the temperature difference and distribution gradient at the molten pool center are substantial; thus, representative points on the transverse cross-section of the cladding layer are analyzed. Reference nodes are selected along the scanning path direction, as illustrated in Figure 4.

**Figure 5:**
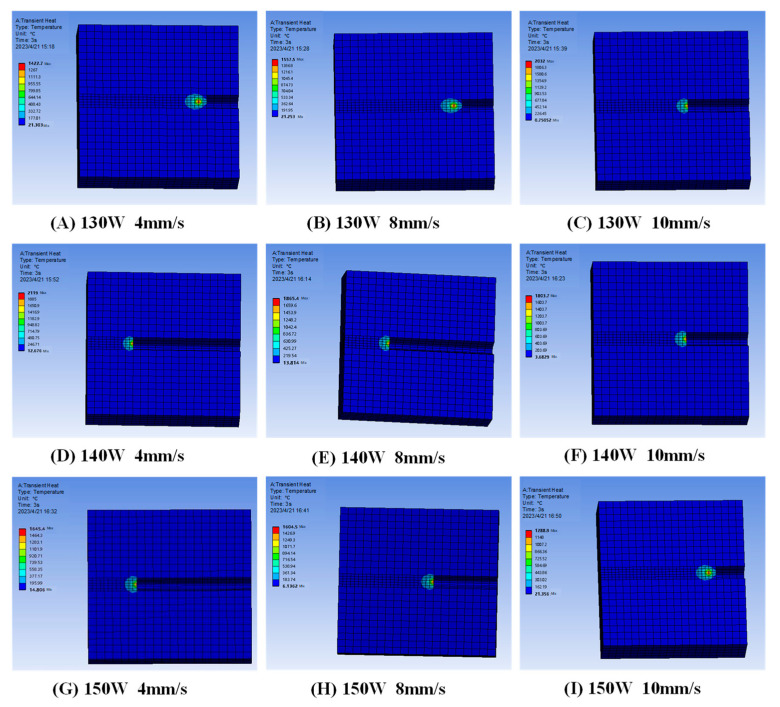
Cloud image of model temperature field under different process parameters.

To investigate the effects of various process parameters on laser cladding formation, the controlled variable method was employed, selecting different combinations of laser power and scanning speed. Nine numerical simulation scenarios were performed under varying process conditions, labeled groups A–I, as shown in Table 2. The temperature field of the model under different process parameters is illustrated in Figure 5.

As shown in groups A, D, and G in Figure 5, the effects of varying laser power on the temperature field are evaluated at a constant scanning speed. When the scanning speed V is set to 4 mm/s, the laser powers P are 130 W, 140 W, and 150 W, and the highest temperatures of the model at the same time are 1654.4 °C, 1865.4 °C, and 2119.0 °C, respectively. At a constant scanning speed, the model’s maximum temperature rises substantially with increasing laser power. This phenomenon is attributed to the increased heat absorption per unit area and time by the substrate, caused by the rising laser energy density, which leads to a noticeable temperature increase.

As seen in groups D, E, and F in Figure 5, the temperature field variations under differing scanning speeds are examined at a constant laser power. When the laser power P is fixed at 140 W, and the scanning speeds are 4 mm/s, 8 mm/s, and 10 mm/s, the model’s maximum temperatures are 1865.4 °C, 1803.7 °C, and 1422.7 °C, respectively. At a constant laser power, the model’s maximum temperature decreases with increasing scanning speed. This reduction occurs because a higher scanning speed reduces the time required to scan the same distance, which decreases accumulated heat, limits substrate heat absorption, and consequently lowers the substrate temperature.

#### 3.1.2. Effect of Different Process Parameters on the Node–Temperature Curve

Initially, the node temperature variation patterns in the first cladding layer under different process parameters are analyzed. In the irradiated region of the cladding layer, a dynamic molten pool is formed under the high-energy laser scan, characterized by rapid heating followed by swift cooling. As the heat source moves, the temperature at each point rises from low to high; upon the heat source’s arrival, the cladding layer temperature increases sharply to a peak, then rapidly declines as the source departs, eventually stabilizing near the model’s average temperature.

**Figure 6:**
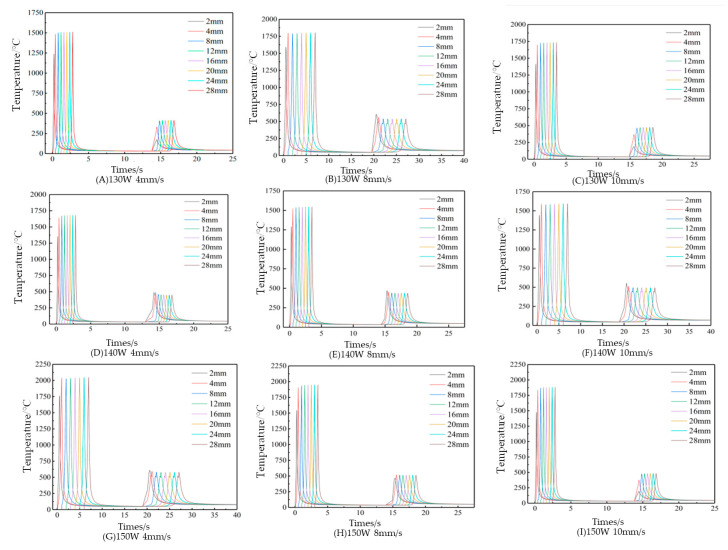
Node temperature–time curve of the first cladding layer under various process parameter combinations.

The impact of laser power on the node–temperature curve of the first cladding layer is analyzed. Based on groups A, D, and G; B, E, and H; or C, F, and I in Figure 6, with a constant scanning speed, the node temperatures rise consistently as the laser power increases. This occurs because the energy absorbed per unit area and time by each node rises, elevating the overall temperatures of both the substrate and cladding layer. Comparing the temperature rise curves reveals a marked increase at higher laser powers; however, excessive laser power could potentially melt through the substrate.

The effect of the scanning speed on the node–temperature curve of the first cladding layer is evaluated. From groups A, B, and C; D, E, and F; or G, H, and I in Figure 6, under constant laser power, an increasing scanning speed results in similar temperature changes across corresponding nodes, but the peak temperatures gradually decrease. This is because faster scanning speeds, for a fixed heat input, allow the laser beam to traverse the substrate more quickly, reducing heat absorption per unit area and time, and thus lowering the peak temperature.

**Figure 7:**
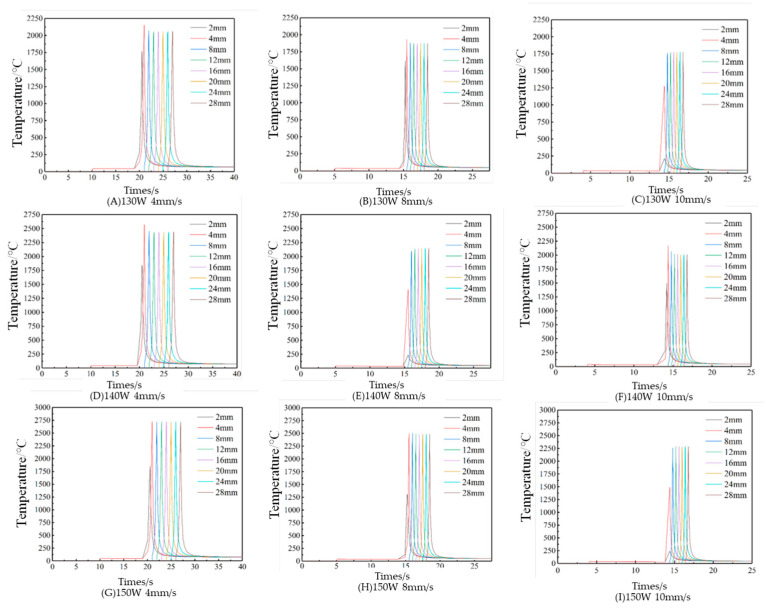
The temperature–time curve of the transverse node of the second cladding layer under different combinations of laser process parameters.

Subsequently, the node temperature variations in the second cladding layer under different process parameters are analyzed. As shown in Figure 7, the temperature variation curves of the transverse nodes in the second cladding layer are generally similar under different laser process parameters, with the temperatures increasing progressively from the Y = 2 mm node and stabilizing near Y = 4 mm. The node temperatures decline rapidly as the heat source departs, resembling the pattern observed in the first cladding layer.

The influence of the laser power on the node–temperature curve of the second cladding layer is examined. From Figure 7, groups A, D, and G; B, E, and H; or C, F, and I indicate that with a constant scanning speed, the node temperatures in the second cladding layer rise as the laser power increases. Compared to the first cladding layer, the second layer exhibits higher temperatures due to a larger defocusing rate, resulting in elevated laser beam power density. The effect of the scanning speed on the second cladding layer is also assessed. In groups A, B, and C; D, E, and F; or G, H, and I in Figure 7, with fixed laser power, node temperatures decrease as the scanning speed increases. Notably, in groups C and I, with a scanning speed of 10 mm/s, the cladding layer temperatures at Y = 2 mm are 241.24 °C and 237.75 °C, respectively. At high scanning speeds, heat absorption inefficiencies may occur at the beginning of the cladding layer.

Through calculations for each model group, the peak temperatures of the temperature fields in the first and second cladding layers under different process parameters were obtained, as shown in Table 3. In laser cladding, the cladding layer temperature must reach the melting points of the substrate material 42CrMo (1410 °C) and the cladding material Fe35 (1700 °C). However, an excessive temperature must be avoided to prevent substrate damage. Based on the data in Table 3, Table 4 and Table 5, when the laser power of P = 140 W and scanning speeds V = 4 mm/s and 8 mm/s, as well as P = 150 W at V = 10 mm/s, the peak temperatures for the first and second cladding layers exceed 1700 °C, but only slightly. At V = 10 mm/s, the temperature variation in the second cladding layer exhibits abnormal heating at the starting position. Therefore, with P = 140 W and scanning speeds between 4–8 mm/s, the temperature field distribution is within the optimal range for cladding. The experimental results indicate that the molten pool shape in the XY direction during laser cladding is influenced by laser power. At powers below 130 W, the alloy powder does not melt uniformly, resulting in an irregular molten pool shape. When the laser power reaches 150 W, the molten pool assumes a nearly elliptical, regular, and smooth distribution.

### 3.2. Analysis of Stress Field Numerical Simulation Results

#### 3.2.1. Effects of Different Process Parameters on Stress Field Contour Plots

**Figure 8:**
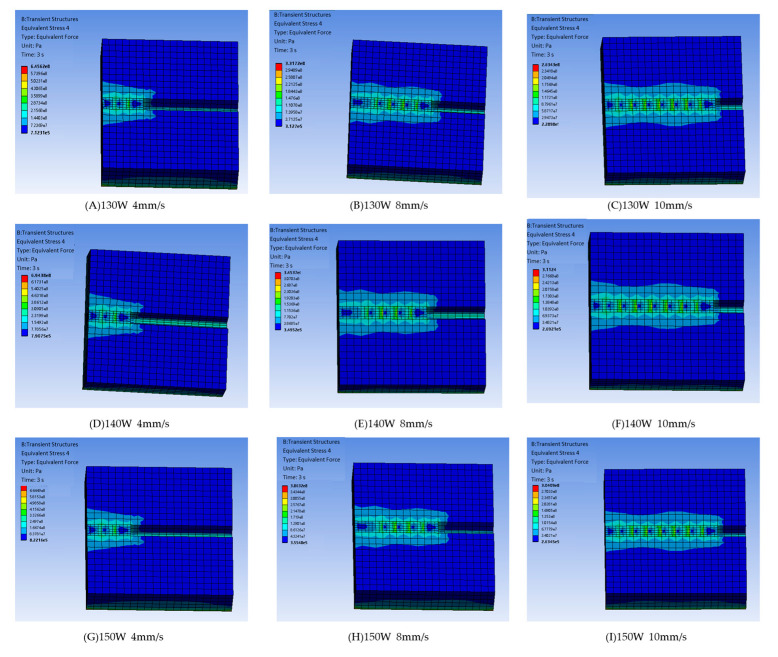
Cloud map of model stress field under different process parameters.

Like the temperature field analysis, by altering the laser power and scanning speed, nine groups of stress field numerical simulation results under different process parameters were obtained, labeled as Groups A–I. Figure 8 shows the 3D stress field contour plots for the nine groups with varying process parameters. Examining the influence of the laser power on the model’s stress field for groups A, D, and G in Figure 8, where the scanning speed is fixed at V = 4 mm/s, and the laser powers are 130 W, 140 W, and 150 W, respectively, the maximum equivalent stresses are 645.62 MPa, 694.38 MPa, and 747.45 MPa. As the laser power increases, the maximum equivalent stress rises significantly due to a higher heat input, which intensifies the temperature gradient between the cladding layer and substrate, generating greater thermal expansion stresses.

Analyzing the effect of the scanning speed on the model’s stress field, as shown in Figure 8, groups D, E, and F, with a constant laser power of P = 140 W and scanning speeds of 4 mm/s, 8 mm/s, and 10 mm/s, the model’s maximum equivalent stresses are 694.38 MPa, 345.37 MPa, and 311.24 MPa, respectively. As the scanning speed increases, equivalent stress across the model reduces progressively due to a lower heat buildup and a smaller temperature gradient. At slower scanning speeds, the increase in equivalent stress is more pronounced. Thus, in practical applications for gear laser cladding repair, increasing the scanning speed can help maintain the cladding layer’s quality.

#### 3.2.2. Effects of Different Process Parameters on Path–Stress Curves

**Figure 9:**
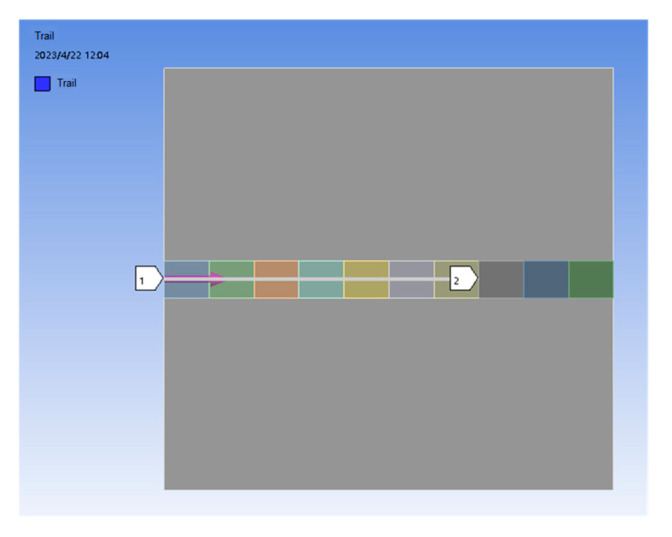
Cladding layer selection path.

The scanning path of the heat source along the transverse surface of the cladding layer is selected as the research subject, starting from the cladding’s initial position 1 and extending to position 2. The model’s selected path is illustrated in Figure 9.

**Figure 10:**
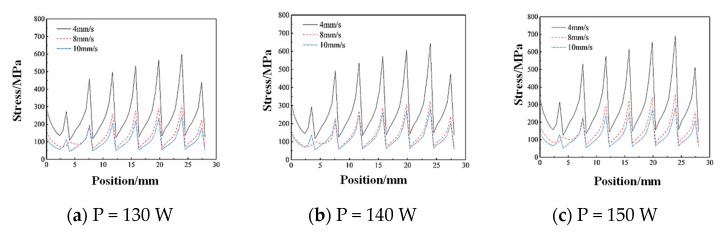
Path–stress variation curves for different scanning speeds at the same power for the first cladding layer.

The stress variation results along this path are retrieved, resulting in the stress variation curves for the first and second layers of the cladding. The stress variation curve for the first layer of the cladding path is presented in Figure 10. Figure 10a shows the effect of the laser power on stress along the first cladding layer path. At a constant scanning speed of V = 4 mm/s and laser powers of 130 W, 140 W, and 150 W, the maximum equivalent stresses are 598.77 MPa, 643.49 MPa, and 693.20 MPa. With higher laser power, the stress values increase consistently. Stress variation along the first cladding layer path is more pronounced than in the second layer, with overall stress values being significantly higher. This is attributed to heat accumulation in the substrate during the first cladding layer, leading to elevated stresses.

Figure 10b illustrates the effect of the scanning speed on stress along the first cladding layer path. With a fixed laser power of P = 140 W and scanning speeds of 4 mm/s, 8 mm/s, and 10 mm/s, the maximum equivalent stresses are 643.49 MPa, 323.96 MPa, and 279.90 MPa. As the scanning speed increases, the stress values decrease consistently. Lower scanning speeds lead to markedly higher stress values due to prolonged heating and a larger temperature gradient.

**Figure 11:**
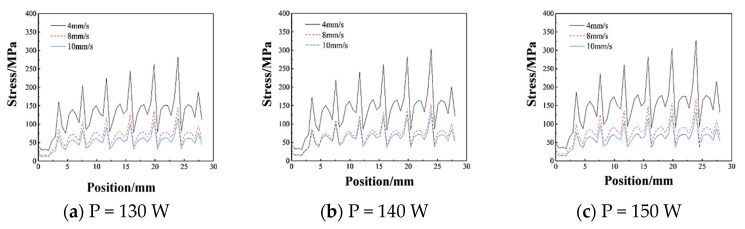
Path–stress variation curves for different scanning speeds at the same power for the second cladding layer.

As shown in Figure 10c, the maximum value of equivalent stress is 693.20 MPa. With the increase in laser power, the value of the stress is increasing. The stress change in the path of the first fused cladding layer is more drastic than that of the second layer, and the overall stress value is higher than that of the second fused cladding layer. This is due to the fact that the substrate is subjected to heat accumulation during the melting of the first layer and hence the stresses are higher as compared to the second layer. The value of stress in the second layer is smaller than in the first layer. The curve for the second layer is illustrated in Figure 11a–c.

By analyzing each model, the maximum equivalent stress values for the first and second layers of cladding under different process parameters were determined and are presented in Table 4. Based on the integrated analyses of the temperature field and stress field numerical simulation data, it was determined that a laser power of P = 140 W and a scanning speed maintained between 4–8 mm/s satisfy the cladding criteria. At a scanning speed of V = 8 mm/s, the maximum equivalent stresses of both the first and second layers of cladding are significantly lower than those observed at the same laser power with a scanning speed of V = 4 mm/s while remaining within the material’s yield limit. According to the finite element numerical simulation results for the temperature and stress fields in laser cladding of gear components, a laser power of P = 140 W and scan speed V = 8 mm/s were identified as optimal theoretical repair parameters.

## 4. Experimental Verification

To validate the cladding performance under the theoretically optimal parameters, the samples were analyzed and tested. Tensile strength was tested using a TK-2T universal material testing machine (Dongguan Zhicheng Instrument Co., Dongguan, China), while hardness was measured using an HV-1000 micro-Vickers tester (Laizhou Lelote Testing Instrument Co., Yantai, China) at nine points across the cladding, heat-affected, and substrate regions at 50 μm intervals under a load of 9.807 N and load time of 10 s. The YH-9600 wear tester (Dongguan Meike Instrument Equipment Co., Dongguan, China) was used in reciprocating the friction mode to conduct wear tests on the cladding layer samples, with a wear volume calculated and the wear morphology of the cladding layer surface observed under an optical magnifier for wear resistance evaluation.

### 4.1. Analysis of Tensile Strength

Tensile tests were conducted using a TK-2T universal material testing machine at a room temperature of 23.3 °C and relative humidity of 51%, in accordance with GB/T 228.1-2021 test standards [23]. The tests aimed to determine the maximum tensile force and tensile strength of the fused cladding layer under nine groups of different laser cladding process parameters and to compare these values with those of the substrate material to analyze the tensile properties of the workpieces after cladding. The specific steps are as follows:

(1) The prepared sample was placed in the fixture of the mechanical testing machine, ensuring alignment between the sample’s long axis and the machine’s axis.
(2) The sample was slightly tensioned prior to the tightening fixture, and the fixture was securely clamped to prevent movement of the test piece.
(3) The specimen was in a substantially unstressed condition before testing.
(4) After balancing the prestressing force, the test speed was set to 5 mm/min, and the test was initiated and continued until the sample fractured. The maximum tensile force of each specimen was recorded.

#### 4.1.1. Analysis of Experimental Data

The influence of the laser power on the cladding layer’s tensile strength was investigated. According to Table 5, at a constant scanning speed of V = 4 mm/s, with laser power settings of 130 W, 140 W, and 150 W, the tensile strengths of the cladding samples were 1029.13 MPa, 1111.67 MPa, and 1194.47 MPa, respectively. The tensile strength of the cladding layer increases with increasing laser power, although excessive laser power leads to unstable tensile strength performance. The influence of the scanning speed on the cladding layer’s tensile properties was examined. According to Table 5, at a fixed laser power of P = 140 W, with scanning speeds of 4 mm/s, 8 mm/s, and 10 mm/s, the tensile strengths of the cladding specimens were 1111.67 MPa, 1312.8 MPa, and 1323.67 MPa, respectively.

The tensile strength of the cladding layer increases with scanning speed. This is largely because the maximum stress the cladding layer experiences decreases with increased scanning speed, leading to an increase in strength, which aligns with the laser cladding stress field numerical simulation results. With optimal process parameters of P = 140 W and V = 8 mm/s, obtained from the laser cladding numerical simulation, the cladding layer’s maximum tensile strength reached 1312.80 MPa, which is 1.22 times the base material’s strength, meeting the repair requirements.

#### 4.1.2. Analysis of Fracture Morphology in the Tensile Test of Cladding Layer

**Figure 12:**
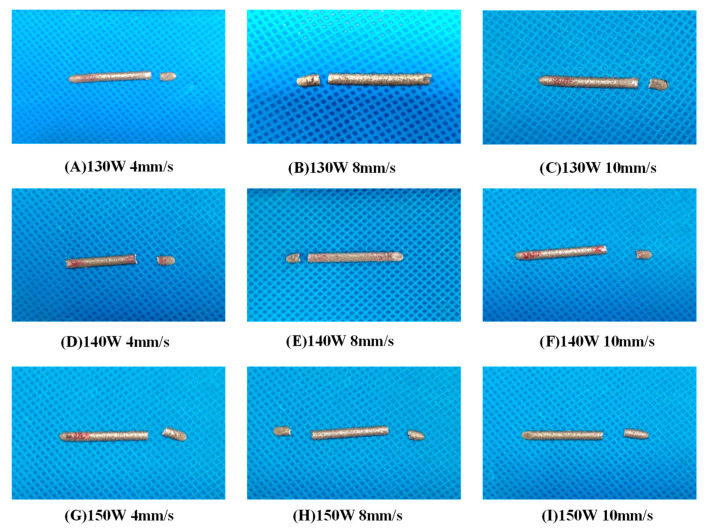
Fracture locations of the cladding layer vary with different process parameters.

Figure 12 shows the macroscopic fracture positions of the tensile test specimens of the cladding layer. The overall image of the fractured tensile specimens indicates that the fractures are located at both ends of the cladding layer, with no significant necking observed and a relatively neat fracture surface. When the laser power is set to P = 150 W and a scanning speed to V = 8 mm/s, the cladding layer shows fractures at both ends.

### 4.2. Analysis of Microhardness

**Figure 13:**
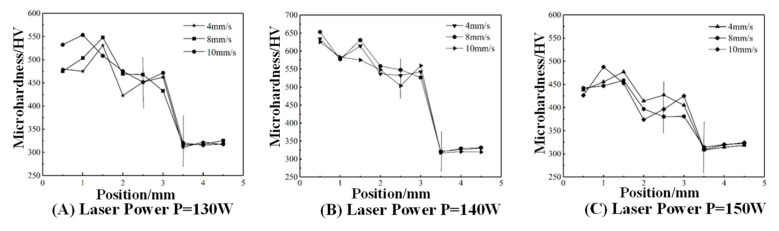
Microhardness of test points taken at different process parameters.

Figure 13 illustrates the effect of the laser power on the microhardness of the cladding layer. At a constant scanning speed of V = 4 mm/s, average microhardness values of 470.33 HV, 573.06 HV, and 436.04 HV were observed at laser powers of 130 W, 140 W, and 150 W, respectively. With increasing laser power, the average microhardness of the cladding layer initially increases and then decreases. This behavior occurs because, at higher laser powers, ample energy per unit of time is provided to the cladding layer surface, enabling sufficient energy absorption by the Fe35 alloy powder, which fully dissolves and achieves good metallurgical bonding with the substrate. However, excessive energy delivered to the cladding layer leads to oxidation and ablation. The excess heat absorption slows cooling, resulting in a coarser internal structure, reduced compactness, and decreased microhardness.

**Figure 14:**
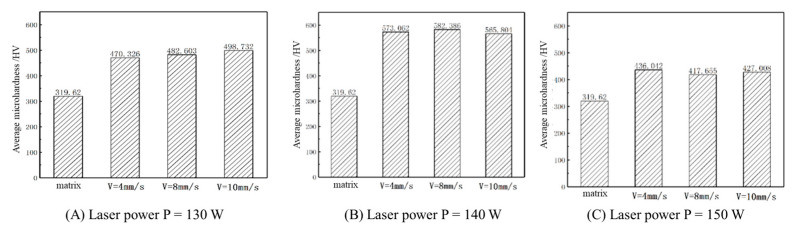
Average microhardness of fused cladding layers with different process parameters.

Figure 14 illustrates the influence of the scanning speed on the microhardness of the cladding layer. At a fixed laser power of P = 130 W, scanning speeds of 4 mm/s, 8 mm/s, and 10 mm/s yielded average microhardness values of 470.33 HV, 482.60 HV, and 498.73 HV, respectively. With an increasing scanning speed, the average microhardness of the cladding layer was observed to increase gradually. Analysis indicates that a moderate increase in scanning speed reduces the contact time between the laser beam and powder, resulting in finer grains and enhanced grain refinement strengthening. Conversely, excessively low scanning speeds prolong contact between the laser beam and powder, promoting coarse grain formation, reduced grain refinement, and lower hardness.

At a laser power of 130 W, dendrites in the cladding layer are large and sparsely distributed. At 140 W, needle-like dendrites with a more uniform distribution appear, and the grain size is reduced. Dendrites become smaller and more compact. When the laser power reaches 150 W, dendrites grow rapidly along the direction of greater heat dissipation, resulting in thinner and coarser structures, leading to reduced hardness.

Under optimal theoretical process parameters, the cladding layer’s microhardness reaches a peak value of 653.29 HV, about 2.05 times the hardness of the substrate at 319.62 HV. Therefore, under these process parameters, the cladding layer shows strong resistance to deformation by rigid objects, reflecting the high quality of the cladding layer.

### 4.3. Abrasion Resistance Analysis

The test instrument used in this abrasion resistance test is a YH-9600 abrasion resistance testing machine; the test temperature is 23.3 °C, and the relative humidity is 51%, according to the test standards, respectively, to detect the wear of the fused cladding layer in nine groups of different laser cladding process parameters in certain loading conditions, and to observe the surface morphology of the fused cladding layer after abrasion. The specific steps are as follows:

(1) Measure the mass m1 of the cleaned specimen with an electronic balance before the wear test.
(2) Fix the specimen on the testing machine. The specimen is in contact with the test ring of 50 mm/min rotational speed and subjected to the weight of 500 g weights. The test is stopped after 5000 revolutions, and the specimen is removed.
(3) After the wear test, the mass m^2^ of the test piece after wear was measured with an electronic balance to derive the mass wear of the test piece, and then an optical magnifying glass was utilized to observe the surface morphology of the fused cladding layer after wear to evaluate the wear resistance of the fused cladding layer.

#### 4.3.1. Analysis of Test Data

**Figure 15:**
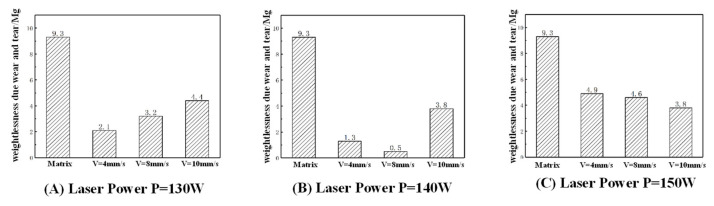
Weight of friction loss for different process parameters.

Figure 15 investigates the influence of the laser power on the wear mass loss of the cladding layer. With a constant scanning speed of V = 4 mm/s and laser powers of 130 W, 140 W, and 150 W, the wear mass loss values are 2.1 mg, 1.3 mg, and 4.9 mg, respectively. As the laser power increases, the wear mass loss decreases initially, then rises. The cross-sectional hardness of the cladding layer initially increases and then decreases. In general, a higher hardness correlates with better wear resistance and reduced wear mass loss. Figure 15 explores the impact of the scanning speed on the wear mass loss in the cladding layer. With a fixed laser power of P = 140 W, scanning speeds of 4 mm/s, 8 mm/s, and 10 mm/s yield wear mass losses of 1.3 mg, 0.5 mg, and 3.8 mg, respectively. As the scanning speed increases, the wear mass loss decreases initially, then rises. When the laser power is P = 140 W, and the scanning speed is V = 8 mm/s, the wear mass loss is minimized, decreasing from 9.3 mg in the substrate to 0.5 mg, achieving high wear resistance and meeting the repair requirements.

#### 4.3.2. Wear Morphology Analysis of Fused Cladding

**Figure 16:**
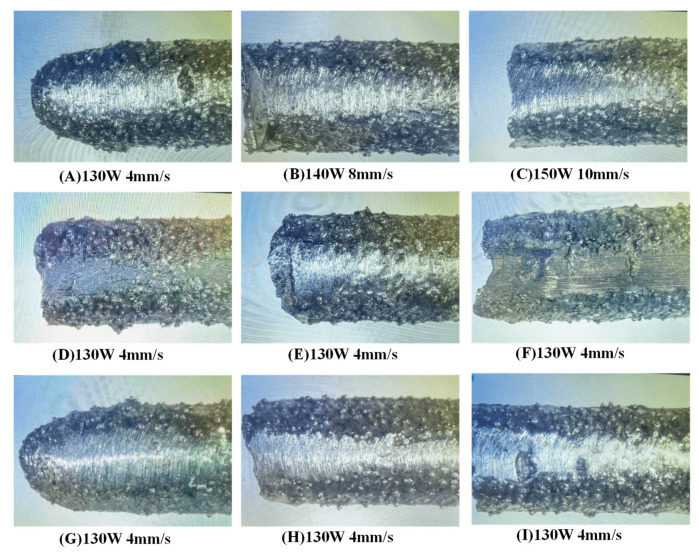
Wear morphology of fused cladding layer under different process parameters.

To further analyze the wear resistance of cladding layers prepared under different process parameters, we chose the same location for the wear test; the surface wear morphology of the cladding layer was observed using an optical microscope, as shown in Figure 16. The figure shows that, under identical friction and wear test conditions, the surfaces of the cladding layers prepared with different process parameters display wear marks of varying degrees. At a laser power of P = 140 W and scanning speed of V = 10 mm/s, the surface wear of the cladding layer is relatively severe, reaching a wear volume of 3.8 mg and exhibiting irregular mechanical damage. In comparison, cladding layers prepared under other process parameters have smoother, flatter wear surfaces with straighter wear marks. At a laser power of P = 140 W and scanning speed of V = 8 mm/s, the wear marks on the cladding layer surface are lighter than those on other surfaces, and the structural integrity of the cladding layer is greatly enhanced. When the laser power is P = 140 W, and the scanning speed is V = 8 mm/s, the wear mass loss reaches its lowest point, reducing from 9.3 mg in the substrate to 0.5 mg, indicating high wear resistance and meeting the repair standards.

## 5. Conclusions

(1) Finite element numerical simulations produced contour plots of the substrate’s temperature and stress fields, analyzing variations in the substrate’s peak temperature and stress under different process parameters. Findings: As the laser power rises, the maximum temperature and stress increase; with higher scanning speeds, both decrease. Analysis of the combined temperature and stress fields concludes that the optimal theoretical process parameters for laser cladding repair of the workpiece are a laser power of P = 140 W and a scanning speed of V = 8 mm/s.
(2) Data from the tensile strength tests indicate that as the laser power and scanning speed rise, so does the cladding layer’s tensile strength. Under the optimal parameters found in laser cladding simulations (P = 140 W, V = 8 mm/s), the maximum tensile strength reaches 1312.80 MPa, or 1.22 times the substrate’s maximum tensile strength, meeting the repair requirements.
(3) The microhardness test data show that the average microhardness of the cladding layer under various process parameters is between 400–600 HV, consistently higher than the 319 HV of the 42CrMo substrate. Also, as the laser power increases, the cladding microhardness rises initially, then drops, while an increased scanning speed raises the average microhardness. At a laser power of P = 140 W and scanning speed of 8 mm/s, the cladding layer’s microhardness peaks at 653.29 HV, about 2.05 times the substrate’s hardness.
(4) The wear resistance test data indicate that cladding layers’ overall wear mass loss under different process parameters is lower than that of the substrate, with the wear mass loss rising initially and then decreasing as the laser power increases. At P = 140 W laser power, the cladding layer shows minimal overall wear mass loss, and with a laser power at P = 140 W and scanning speed of V = 8 mm/s, the wear mass loss reaches a minimum, reducing from the substrate’s 9.3 mg to 0.5 mg, indicating high wear resistance.

## Figures and Tables

**Table 1:** Other thermal properties of the material.

Material	Density/kg/m^3^	Melting Point/K	Coefficient of Thermal Expansion/K^−1^	Modulus of Elasticity/GPa	Poisson Ratio
42CrMo	7850	1683	1.1 × 10^−5^	212	0.28
Fe35	6250	1973	3.42 × 10^−5^	212	0.32

**Table 2:** Other thermal properties of the material.

Group Number	Laser Power (P/W)	Scanning Speed (V/mm/s)
A	130	4
B	130	8
C	130	10
D	140	4
E	140	8
F	140	10
G	150	4
H	150	8
I	150	10

**Table 3:** Other thermal properties of the material.

Group	Laser Power/W	Scanning Speed/mm/s	Highest Temperature of the First Cladding Layer/°C	Highest Temperature of the Second Cladding Layer/°C
A	130	4	1592.6	2059.3
B	130	8	1544.1	1872.3
C	130	10	1508.8	1771.6
D	140	4	1803.0	2438.1
E	140	8	1733.3	2147.3
F	140	10	1680.4	2007.7
G	150	4	2046.8	2718.4
H	150	8	1948.9	2483.0
I	150	10	1881.6	2288.1

**Table 4:** Maximum equivalent stress values for the model stress field across different process parameters.

Group	Laser Power/W	Scanning Speed/mm/s	Maximum Equivalent Force of the First Cladding Layer/MPa	Maximum Equivalent Force of the Second Cladding Layer/MPa
A	130	4	655.63	374.60
B	130	8	333.94	191.11
C	130	10	265.49	152.20
D	140	4	705.24	402.73
E	140	8	352.52	202.21
F	140	10	305.55	174.29
G	150	4	760.06	433.69
H	150	8	388.80	221.78
I	150	10	306.60	175.72

**Table 5:** Maximum tensile force and tensile strength results of samples.

Group	Laser Power/W	Scanning Speed/mm/s	Maximum Tensile Force/N	Tensile Strength/MPa
A	130	4	1543.7	1029.13
B	130	8	1901.6	1267.73
C	130	10	1905.3	1270.20
D	140	4	1667.5	1111.67
E	140	8	1969.2	1312.80
F	140	10	1985.5	1323.67
G	150	4	1791.7	1194.47
H	150	8	1855.5	1237.00
I	150	10	2144.4	1429.60

## Data Availability

The raw data supporting the conclusions of this article will be made available by the authors upon request.
